# Two Kinds of Ferritin Protect Ixodid Ticks from Iron Overload and Consequent Oxidative Stress

**DOI:** 10.1371/journal.pone.0090661

**Published:** 2014-03-03

**Authors:** Remil Linggatong Galay, Rika Umemiya-Shirafuji, Eugene T. Bacolod, Hiroki Maeda, Kodai Kusakisako, Jiro Koyama, Naotoshi Tsuji, Masami Mochizuki, Kozo Fujisaki, Tetsuya Tanaka

**Affiliations:** 1 Department of Pathological and Preventive Veterinary Science, The United Graduate School of Veterinary Science, Yamaguchi University, Yoshida, Yamaguchi, Japan; 2 Laboratory of Emerging Infectious Diseases, Joint Faculty of Veterinary Medicine, Kagoshima University, Korimoto, Kagoshima, Japan; 3 National Research Center for Protozoan Diseases, Obihiro University of Agriculture and Veterinary Medicine, Inada-cho, Obihiro, Hokkaido, Japan; 4 The United Graduate School of Agricultural Sciences, Kagoshima University, Shimoarata, Kagoshima, Japan; 5 Department of Chemistry, College of Arts and Sciences, University of San Carlos, Cebu City, Philippines; 6 Education and Research Center for Marine Resources and Environment, Faculty of Fisheries, Kagoshima University, Shimoarata, Kagoshima, Japan; 7 National Agricultural and Food Research Organization, Kannondai, Tsukuba, Ibaraki, Japan; Onderstepoort Veterinary Institute, South Africa

## Abstract

Ticks are obligate hematophagous parasites that have successfully developed counteractive means against their hosts' immune and hemostatic mechanisms, but their ability to cope with potentially toxic molecules in the blood remains unclear. Iron is important in various physiological processes but can be toxic to living cells when in excess. We previously reported that the hard tick *Haemaphysalis longicornis* has an intracellular (HlFER1) and a secretory (HlFER2) ferritin, and both are crucial in successful blood feeding and reproduction. Ferritin gene silencing by RNA interference caused reduced feeding capacity, low body weight and high mortality after blood meal, decreased fecundity and morphological abnormalities in the midgut cells. Similar findings were also previously reported after silencing of ferritin genes in another hard tick, *Ixodes ricinus*. Here we demonstrated the role of ferritin in protecting the hard ticks from oxidative stress. Evaluation of oxidative stress in *Hlfer*-silenced ticks was performed after blood feeding or injection of ferric ammonium citrate (FAC) through detection of the lipid peroxidation product, malondialdehyde (MDA) and protein oxidation product, protein carbonyl. FAC injection in *Hlfer*-silenced ticks resulted in high mortality. Higher levels of MDA and protein carbonyl were detected in *Hlfer*-silenced ticks compared to *Luciferase*-injected (control) ticks both after blood feeding and FAC injection. Ferric iron accumulation demonstrated by increased staining on native HlFER was observed from 72 h after iron injection in both the whole tick and the midgut. Furthermore, weak iron staining was observed after *Hlfer* knockdown. Taken together, these results show that tick ferritins are crucial antioxidant molecules that protect the hard tick from iron-mediated oxidative stress during blood feeding.

## Introduction

Iron is an essential element required for various physiological processes in most living organisms. Iron metabolism involves a continuous redox cycling between the ferrous (Fe^2+^) and ferric (Fe^3+^) states. Fe^2+^ is potentially toxic due to its ability to catalyze the formation of reactive oxygen species (ROS) through Fenton reaction [1]. High levels of ROS can lead to cellular damage and death, resulting from damage to biomolecules including lipid peroxidation, DNA and protein oxidation, which is collectively known as oxidative stress [2]. Oxidative stress occurs when the level of ROS overwhelms the antioxidant defense mechanisms, accompanied by the accumulation of oxidative stress products. These products of oxidative damage to biomolecules can be used as indicators in evaluating oxidative stress, termed biomarkers [3].

Iron-binding proteins, such as transferrin and ferritin, are present in most living organisms that function to regulate iron levels and prevent iron toxicity. Most ferritins consist of 24 subunits folded in a helical bundle, forming an almost spherical protein shell with a large cavity that can hold up to 4,000 iron atoms [4]. Mammalian ferritins serve mainly as intracellular iron storage proteins, while insect ferritins also function in iron transport [5]. Aside from iron transport and storage functions, ferritin was also implicated in immune response [6] and oxidative stress [7].

Ticks are important blood-feeding parasites of wild and domestic animals and humans, primarily because they serve as vectors of different pathogens. Aside from dealing with the host's hemostatic and immune mechanism [8], ticks must also cope with the potentially toxic molecules in their large blood meal, including iron. However, many aspects of iron metabolism of ticks remain unclear. Heme transport [9], [10] and detoxification [11] have already been investigated. An intracellular and a secretory ferritin in two species of hard ticks, *Ixodes ricinus* [12] and *Haemaphysalis longicornis* [13] have been reported to be crucial in blood feeding and reproduction. The other functions of ferritin, particularly its role in tick survival, have not yet been fully elucidated. Existing knowledge on the antioxidant defense of ticks, especially during blood feeding, is limited.

Here we showed that RNA interference (RNAi)-mediated silencing of *H. longicornis* ferritin genes predisposed the ticks to oxidative stress by detecting the levels of a product of lipid peroxidation and a product of protein oxidation after blood feeding or iron injection. Our results show that the two ferritins of *H. longicornis* are essential antioxidant molecules that prevent iron-mediated oxidative stress during blood feeding and are crucial to its survival.

## Materials and Methods

### Ticks and experimental animals

Parthenogenetic (Okayama strain) adult female *H. longicornis* ticks were used throughout this study. Ticks have been maintained by feeding on the ears of Japanese white rabbits (Kyudo, Kumamoto, Japan) for several generations at the Laboratory of Emerging Infectious Diseases, Joint Faculty of Veterinary Medicine, Kagoshima University, Kagoshima, Japan [14]. Rabbits were kept in a temperature- and humidity-controlled room, with a constant supply of water and commercial rabbit pellets. Rabbit care and use in this study has been approved by the Animal Care and Use Committee of Kagoshima University (Approval number VM13007).

### RNA interference and tick infestation

The silencing of *Hlfer* in unfed adult female ticks was induced by injection of double-stranded RNA (dsRNA) prepared as previously described [13]. Briefly, ticks were attached to glass slides and then injected with 1 µg per 0.5 µl of *Hlfer1* or *Hlfer2* dsRNA through the fourth coxae using an IM 300 Microinjector (Narishige, Tokyo, Japan). Control ticks were injected with the same amount of firefly *Luciferase* (*Luc*) dsRNA. To confirm silencing, total RNA was extracted from whole ticks 4 days post-injection of dsRNA for RT-PCR analysis. Ticks injected with dsRNA were held in a humidity chamber kept in a 25°C incubator for 18 h before infestation to rabbits or for 4 days before injection with ferric ammonium citrate (FAC).

For rabbit infestation, a total of 50 ticks per dsRNA injected group were attached in separate ears of rabbits, individually covered with an ear bag. Attached ticks were allowed to feed until they naturally dropped off. From the total number of engorged ticks, 30 ticks from each group were used for the thiobarbituric acid reactive species (TBARS) assay in the whole ticks. Five pooled midgut samples, comprising of three ticks each for *Hlfer1*and *Hlfer2*-silenced ticks and two ticks each for *Luc*-injected group, were also prepared for the TBARS assay. The remaining ticks were used for immunoblot detection of oxidative stress biomarkers, described in the succeeding sections. All ticks were stored in −80°C until use.

### Injection of ferric ammonium citrate (FAC)

We previously found that the silencing of either *Hlfer1* or *Hlfer2* had a negative effect on tick survival after blood feeding [13] and we concluded that this was caused by iron overload. Thus, to further investigate the effect of high levels of iron on ticks, different concentrations of FAC were injected into unfed adult ticks, with or without dsRNA injection. To check the survival rate of *Hlfer*-silenced ticks after exposure to iron, 100 µM FAC was injected in the same manner as dsRNA injection. Likewise, sterilized high-purity water was injected to dsRNA-injected ticks for additional control. Thirty ticks for each group were used for this experiment. After injection of FAC, ticks were held as mentioned above and monitored for mortality every 12 h for 11 days. The survival experiment was repeated three times to confirm the reproducibility of results. Otherwise, unfed adult ticks not injected with dsRNA were injected with 50 or 100 µM FAC or sterilized high-purity water for control to evaluate mRNA and protein expression and iron staining in response to iron treatment.

### Protein extraction

Blood-fed or FAC-injected whole ticks were homogenized in phosphate-buffered saline (PBS). Midguts and salivary glands were also collected and homogenized in Tris-buffered saline (TBS) with a protease inhibitor (Complete Mini EDTA-free, Roche, Manheim, Germany). Hemolymph was collected from the amputated legs of immobilized ticks. Hemocytes were separated by centrifugation. Protein from whole ticks and organs was extracted as previously described [15]. Protein samples were kept at −80°C until use.

### Electrophoresis, Western blot analysis and gel iron staining

To investigate the protein expression, protein samples were separated in 12% SDS-polyacrylamide gel electrophoresis (PAGE) and subjected to Western blot analysis as described previously [13]. Specific mouse anti-ferritin sera [13] or anti-β-tubulin serum for control [16] were used as primary antibodies. Protein signals were detected using the ECL Prime Western Blotting Detection Reagent (GE Healthcare, Little Chalfont, Buckinghamshire, UK) and images were taken using the FluorChem FC2 Imaging System (Protein Simple, Santa Clara, CA, USA). Western blotting was performed at least three times. To accurately determine differences in the protein expression, band densitometry analysis was performed using Alpha View Software (Alpha Innotech, Protein Simple). The band densitometry analysis results shown in this study represent the mean of three trials of Western blot analysis.

To stain native HlFER for ferric iron, protein extracts were separated in 6% native PAGE. Protein concentration was adjusted after determination of protein concentration using a Micro BCA Assay kit (Thermo Scientific, Rockford, IL, USA) or based on control immunoblotting with β-tubulin as described above [16]. The gel was stained in a freshly prepared Prussian blue staining solution (equal volume of 10% K_4_[Fe(CN)_6_] and 10% HCl) at room temperature for 48 h as previously reported [17]. The high molecular weight marker (GE Healthcare), which contains ferritin from equine spleen for the 440 kDa band, as well as the commercially-prepared horse holoferritin (Sigma-Aldrich, St. Louis, MO, USA) were used as positive controls.

### Immunofluorescent examination of organs after FAC injection

An indirect immunofluorescent antibody test (IFAT) was performed as previously described [13]. Briefly, midguts and salivary glands were dissected from unfed adult ticks then fixed overnight in 4% paraformaldehyde in PBS with 0.1% glutaraldehyde and washed with a sucrose series before being embedded in Tissue-Tek O.C.T Compound (Sakura Finetek Japan, Tokyo, Japan). After cutting, tissue sections were air-dried and then blocked overnight with 5% skim milk in PBS at 4°C. Sections were incubated with a 1∶50 dilution of anti-ferritin sera or normal mouse serum as a control for the primary antibody and a 1∶1,000 dilution of Alexa Fluor 594-conjugated goat anti-mouse IgG (Invitrogen, Eugene, OR, USA) for the secondary antibody for an hour each at room temperature. Following washes with PBS, sections were mounted in Vectashield with DAPI (Vector Laboratories, Burlingame, CA, USA) and then viewed on a fluorescence microscope mounted with a DP71 camera (Olympus, Tokyo, Japan).

### Assessment of oxidative stress

Oxidative stress was evaluated by detecting oxidative stress biomarkers including malondialdehyde (MDA) and protein carbonyl (PC). MDA was demonstrated through immunoblotting using the Oxiselect Malondialdehyde Immunoblot Kit (Cell Biolabs, San Diego, CA, USA) following the manufacturer's recommendation. Engorged whole ticks were homogenized individually, whereas midguts and unfed adult ticks injected FAC were pooled. Protein was adjusted based on tubulin profile. Bands were viewed using Clarity Western ECL Substrate (Bio-rad Laboratories, Hercules, CA, USA) and the MDA level relative to tubulin was calculated after band densitometry analysis. TBARS assay was also performed to quantify MDA [18]. For the TBARS assay, tick homogenates were mixed with TBARS reagent (0.37% (w/v) thiobarbituric acid, 15% (w/v) trichloroacetic acid in 0.25 M HCl) and then placed in boiling water bath for 15 min and allowed to cool. Absorbance was measured at 532 nm and MDA content was calculated using the molecular extinction coefficient for MDA. PC was also demonstrated following the immunoblot assay using the Oxiselect Protein Carbonyl Immunoblot Kit (Cell Biolabs) according to the manufacturer's instruction and analyzed similar to MDA.

### Measurement of total ferrous iron

The ferrozine assay for measuring non-heme iron was adapted to determine the amount of ferrous iron in whole ticks injected with FAC after *Hlfer* knockdown [19], [20]. Ten whole ticks from each group were collected 72 h after the injection of FAC and homogenized in lysis buffer (20 mM Tris, 137 mM NaCl, 1% Triton X-100, 1% glycerol). Protein concentration was measured using a Micro BCA Assay Kit (Thermo Scientific). Concentrated HCl was added and then heated to 95°C. After cooling to room temperature, the mixture was centrifuged and the supernatant was obtained, to which 10 mM ferrozine was added. Color development was accomplished by the addition of saturated ammonium acetate. Absorbance was measured at 550 nm and iron concentration was calculated based on a molar extinction coefficient of the iron-ferrozine complex of 27 900 M^−1^ cm^−1^ and based on protein concentration.

### Statistical analyses

For band densitometry analysis, Student's *t*-test or the Mann-Whitney U test was performed, depending on data distribution. For the survival experiment, the Mantel-Cox log-rank test was performed using GraphPad Prism software. In all statistical analyses, significant difference between groups is defined by *P*<0.05.

## Results

### *Hlfer*-silenced ticks had low survival rate after FAC injection

**Figure 1:**
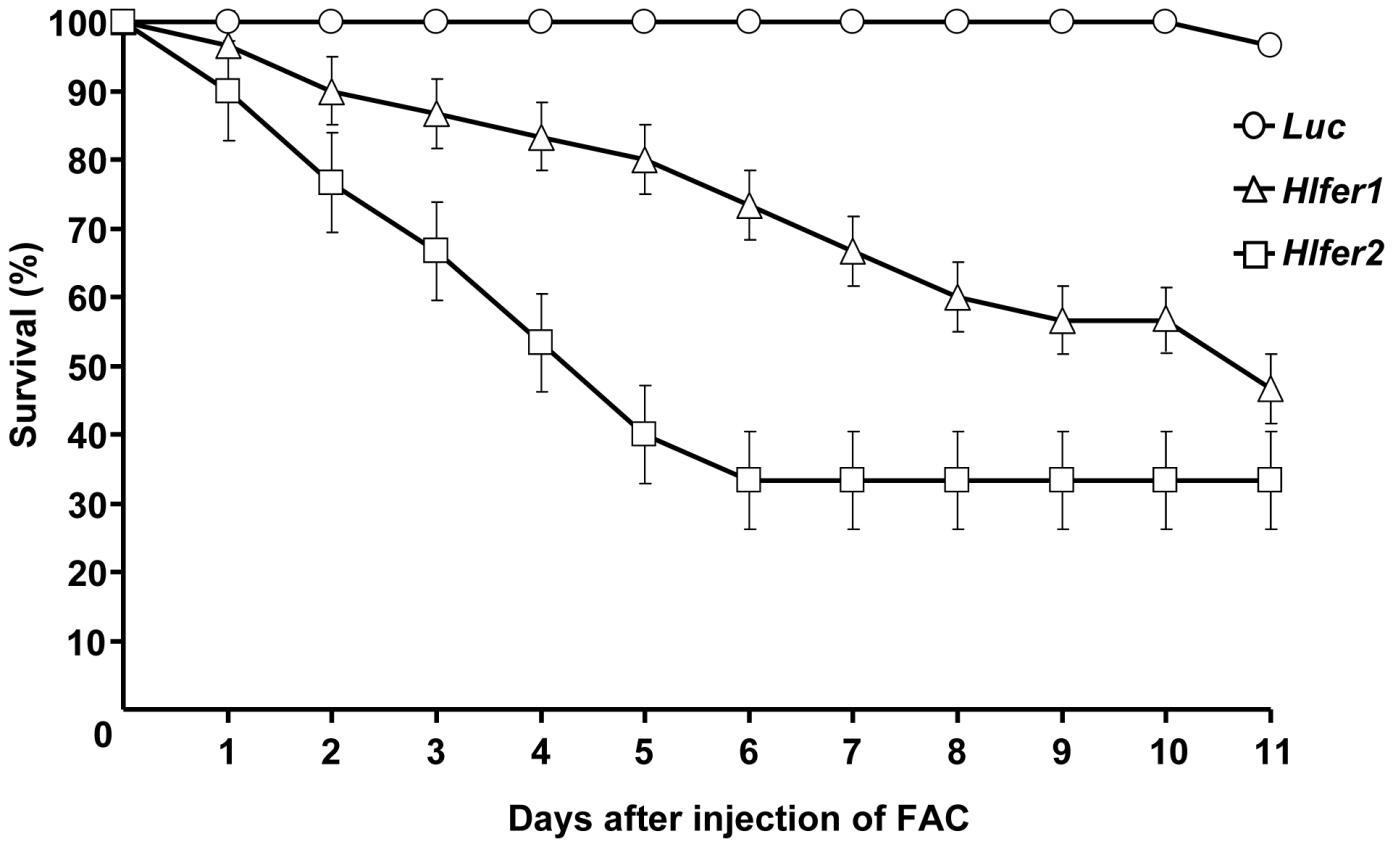
Survival rate of *Hlfer*-silenced ticks after injection of FAC. Unfed adult female ticks were injected with *H. longicornis fer1* (*Hlfer1*), *H. longicornis fer2* (*Hlfer2*) or *Luciferase* (*Luc*) dsRNA for the control to induce RNAi. Silencing was confirmed through RT-PCR. After 4 days, 100 µM FAC was injected, and mortality was monitored. Both *Hlfer1*- and *Hlfer2*-dsRNA-injected groups had a lower survival rate compared to *Luc*. n = 30 ticks per group. The graph here represents the result of a single independent trial. Bars represent standard error. Significant difference was determined using the log-rank Mantel-Cox test (*P*<0.0001, *Luc* vs. *Hlfer1* or *Hlfer2*).

High mortality was previously observed in *Hlfer*-silenced ticks after blood feeding [13]. To further demonstrate that the low survival rate was related to iron overload in the absence of ferritin, here we exposed unfed *Hlfer*-silenced adult female ticks to iron by injecting 100 µM FAC into the hemocoel. Silencing was confirmed by RT-PCR analysis (data not shown). After FAC injection, ticks were kept at 25°C and survival was monitored every 12 h. No mortality was observed in the control group injected with *Luc* dsRNA (Fig. 1). In contrast, both *Hlfer1*- and *Hlfer2*-silenced groups had a continuously decreasing survival rate (*P*<0.0001). Eleven days after FAC injection, the *Hlfer2* dsRNA-injected group showed the lowest survival rate. As an additional negative control, high-purity sterilized water was similarly injected after RNAi but this did not result in high mortality as in the case of FAC injection (Fig. S1). This result supports our previous conclusion that the mortality after blood feeding in *Hlfer*-silenced ticks was due to iron toxicity.

### FAC injection has no effect on transcription but stimulates protein expression of ferritins

**Figure 2:**
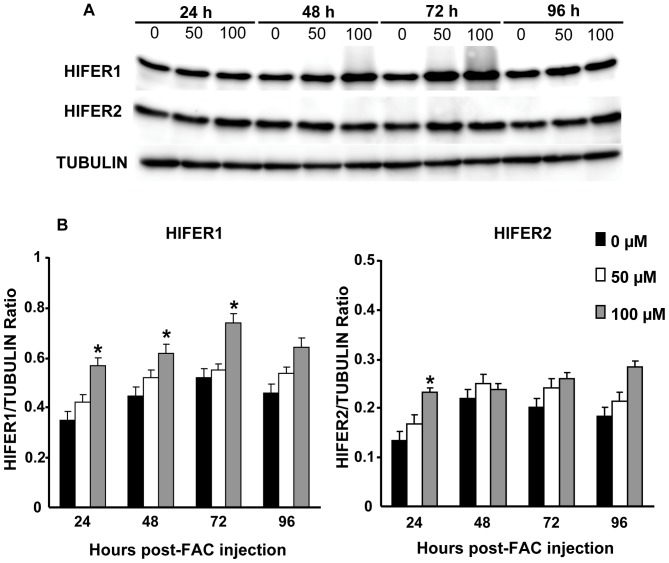
Protein expression of *H. longicornis* ferritins in whole, unfed ticks at different hours after injection of different concentrations of FAC. Sterilized high-purity water was injected into the control group (0 µM). (A) Western blot analysis after incubation with specific anti-sera against *H. longicornis* FER1 (HlFER1) or *H. longicornis* FER2 (HlFER2). Tubulin was used as an internal control. (B) Band densitometry analysis for HlFER1 and HlFER2. The relative expression was calculated based on tubulin. Significant increase in expression was particularly found in HlFER1. Data represent the means of three independent trials ± SE. Statistical significance (*P<0.05) was determined using the Mann-Whitney test.

**Figure 3:**
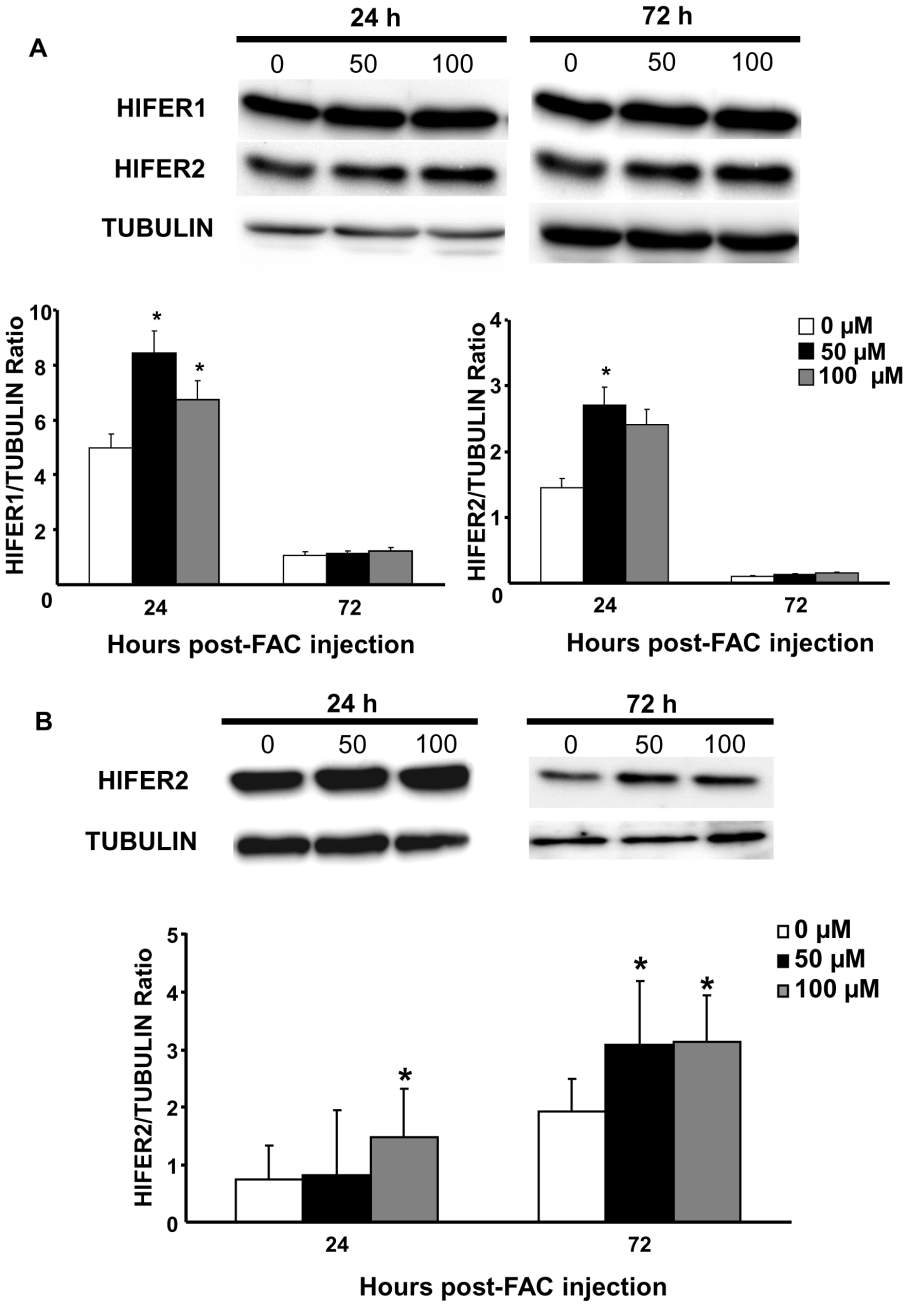
Protein expression of *H. longicornis* ferritins in midguts (A) and hemolymph (B) of unfed ticks injected with different concentrations of FAC. Midguts and hemolymph were collected at 24 µM or 100 µM FAC or sterilized high purity water for the control group (0 µM). Hemocyte was separated from the hemolymph by centrifugation. Western blot analysis was performed using specific anti-sera against *H. longicornis* FER1 (HlFER1) or *H. longicornis* FER2 (HlFER2). Tubulin was used as an internal control. The relative expression of HlFER1 and HlFER2 was calculated based on tubulin after band densitometry analysis. Significant increase in expression was particularly found in HlFER1. Data represent the means of three independent trials ± SE. Statistical significance (*P<0.05) was determined using the Mann-Whitney test.

We evaluated whether injection of FAC as exogenous iron source can affect *Hlfer* transcript level and HlFER protein expression. Artificial feeding or *in vitro* exposure of cells to iron in different organisms induced up-regulation of ferritin mRNA [21], [22], [23], [24], [25]. Different concentrations of FAC were injected to the hemocoel of normal unfed adult female ticks or sterilized high-purity water for the control group. The transcript level in whole ticks was then checked at 24 h and 72 h after FAC injection, whereas protein expression was examined from 24 h to 96 h after FAC injection. RT-PCR analysis showed no difference among the groups at any time point (Fig. S2). However, increased protein expression particularly of HlFER1 was observed in both concentrations of FAC from 24 h to 96 h post-injection (Fig. 2). Band densitometry analysis was performed to accurately determine the differences in protein expressions. We also examined the HlFER expression in organs at 24 h and 72 h post-injection and we found that both HlFER1 and HlFER2 levels were higher in the midguts (Fig. 3A) of FAC injected ticks but not in the salivary glands (Fig. S3). In the hemolymph where only HlFER2 is present, its expression is also higher after FAC injection compared to the control (Fig. 3B). These findings suggest that iron injection can stimulate HlFER expression in unfed ticks.

### FAC injection led to ferric iron accumulation in HlFER in the whole tick and the midgut

**Figure 4:**
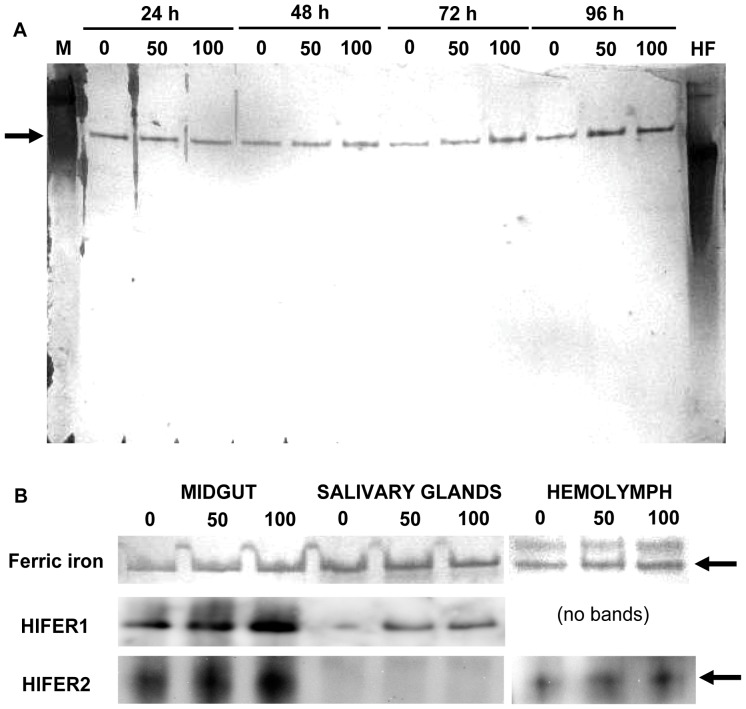
Staining of HlFER for ferric iron on native PAGE gel after FAC injection. Ticks were injected with 50 or 100 µM FAC or sterilized high-purity water (0 µM) for the control group. Whole ticks were collected from 24 h to 96 h and total protein was extracted (A). Each lane of native PAGE gel was loaded with 20 µg of total protein and then the gel was stained with an equal volume of 10% K_4_[Fe(CN)_6_] and 10% HCl for up to 48 h. A single band stained for ferric iron. The high molecular weight marker (M), which contains ferritin from equine spleen, also stained for ferric iron, as well as the horse holoferritin (HF), used as positive control. Midguts, salivary glands, and the hemolymph (B) were also collected from ticks 72 h after injection of FAC or sterilized high-purity water. Each lane was loaded with 10 µg of protein. Western blot analysis using specific anti-HlFER sera was also performed to confirm that the band stained for ferric iron is HlFER. Arrows indicate approximately 440 kDa, which is the theoretical molecular weight of native ferritin.

After observing that FAC can stimulate HlFER expression in the whole tick and in the midgut, we determined whether there was a corresponding accumulation of ferric iron on native HlFER. After separating the tick protein in native PAGE, HlFER was stained using Prussian blue staining to indicate ferric iron. Both the high molecular weight marker containing ferritin from equine spleen as the 440 kDa band, and the commercial horse holoferritin strongly stained for ferric iron (Fig. 4A). In whole ticks, increased staining was observed at 72 h and 96 h after injection of any concentration of FAC (Fig. 4A). Ferric iron staining also increased in the midgut and hemolymph at 72 h post-injection of FAC but not in the salivary glands (Fig. 4B) or ovary (data not shown). In all experiments, only one band was stained with Prussian blue, with an estimated molecular weight of around 440 kDa. We confirmed that the bands stained for ferric iron were HlFERs through Western blot analysis after native PAGE (Fig. S4 and 4B). HlFER1 and HlFER2 had almost the same molecular weight on native PAGE.

### FAC injected into the hemocoel stimulated HlFER expression of digestive cells in the midgut

**Figure 5:**
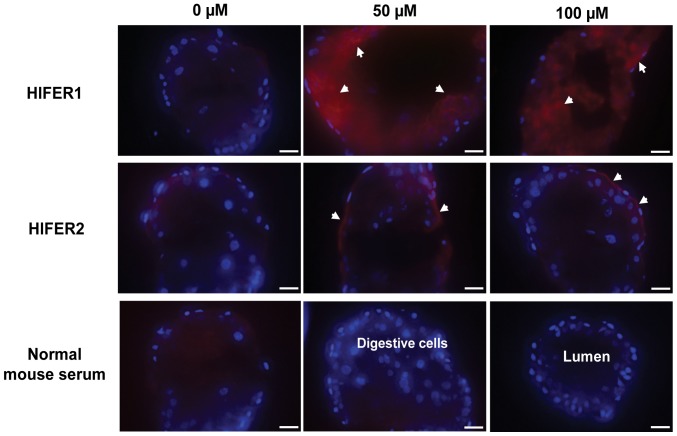
An indirect immunofluorescent antibody test (IFAT) in the midgut after injection of FAC. Different concentrations of FAC or sterilized high-purity water (0 µM) were injected to unfed adult ticks and midguts were collected after 72 h. To visualize the extent of the stimulation of HlFER expression caused by FAC injection, frozen sections of the midgut were incubated with specific mouse anti-HlFER1 or anti-HlFER2 sera. Mouse normal serum was used as a negative control. Anti-mouse IgG conjugated with Alexa 594 was used as secondary antibody and nuclei were visualized using DAPI. Arrowheads point to areas with increased fluorescence. (Bars  = 20 µm).

It is interesting that injection of FAC to the hemocoel stimulated HlFER expression in the midgut, as shown by Western blot analysis, and that the midgut can also store the iron from the hemolymph, as demonstrated by ferric iron staining on native PAGE. We wanted to know the extent of the effect of FAC on HlFER expression of digestive cells, therefore we performed IFAT. The salivary gland was also examined for comparison. Midguts and salivary glands were collected from normal unfed adult female ticks 72 h after injection of FAC or sterile high-purity water. Increased fluorescence was observed in digestive cells 72 h after injection of 50 µM and 100 µM FAC (Fig. 5). For HlFER1, extensive fluorescence was observed throughout the midgut, from the basal lamina up to the inner digestive cells lining the lumen. For HlFER2, much of the fluorescence was observed along the basal lamina. In contrast, very weak fluorescence for both HlFERs was observed in the salivary glands (Fig. S5).

### *Hlfer-*silenced ticks had higher levels of oxidative stress biomarkers after blood feeding or FAC injection

Iron is known to catalyze the formation of ROS in living cells, thus promoting oxidative stress. We previously found that *Hlfer*-silenced ticks had abnormal midgut morphology and high mortality after blood feeding and we hypothesized that this was caused by oxidative stress. Thus, we evaluated the oxidative status of *Hlfer*-silenced ticks after blood feeding or exposure to exogenous iron through demonstration of known oxidative stress biomarkers. We detected malondialdehyde (MDA), a known product of lipid peroxidation [3], and protein carbonyl (PC) resulting from the oxidation of proteins [26].

**Figure 6:**
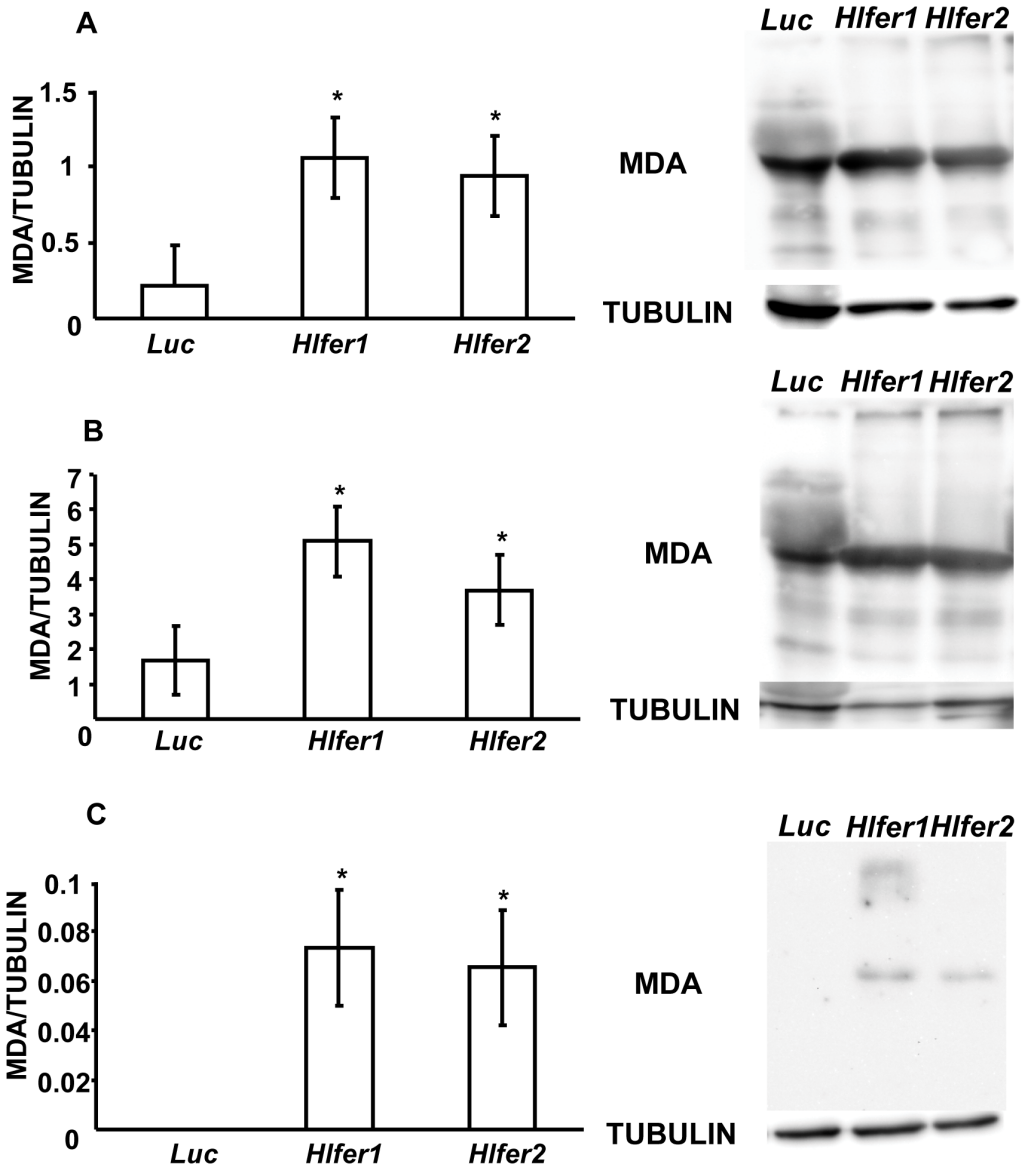
Detection of malondialdehyde (MDA) from *Hlfer*-silenced ticks after blood feeding or injection of FAC. Total protein was extracted from whole ticks (A) and midguts (B) after blood feeding and whole ticks 72 h after injection of 100 µM FAC (C). Western blot analysis was performed and the membrane was incubated with a specific anti-MDA antibody. Tubulin was used as internal control. The relative content of MDA (clearest band) to tubulin was calculated after band densitometry analysis. Both *Hlfer1*- and *Hlfer2*-silenced ticks had significantly higher MDA compared to the control (*Luc*) group. Data represent the means of three independent trials ± SE. *P<0.05, significantly different vs. *Luc*, Student's *t*-test.

**Figure 7:**
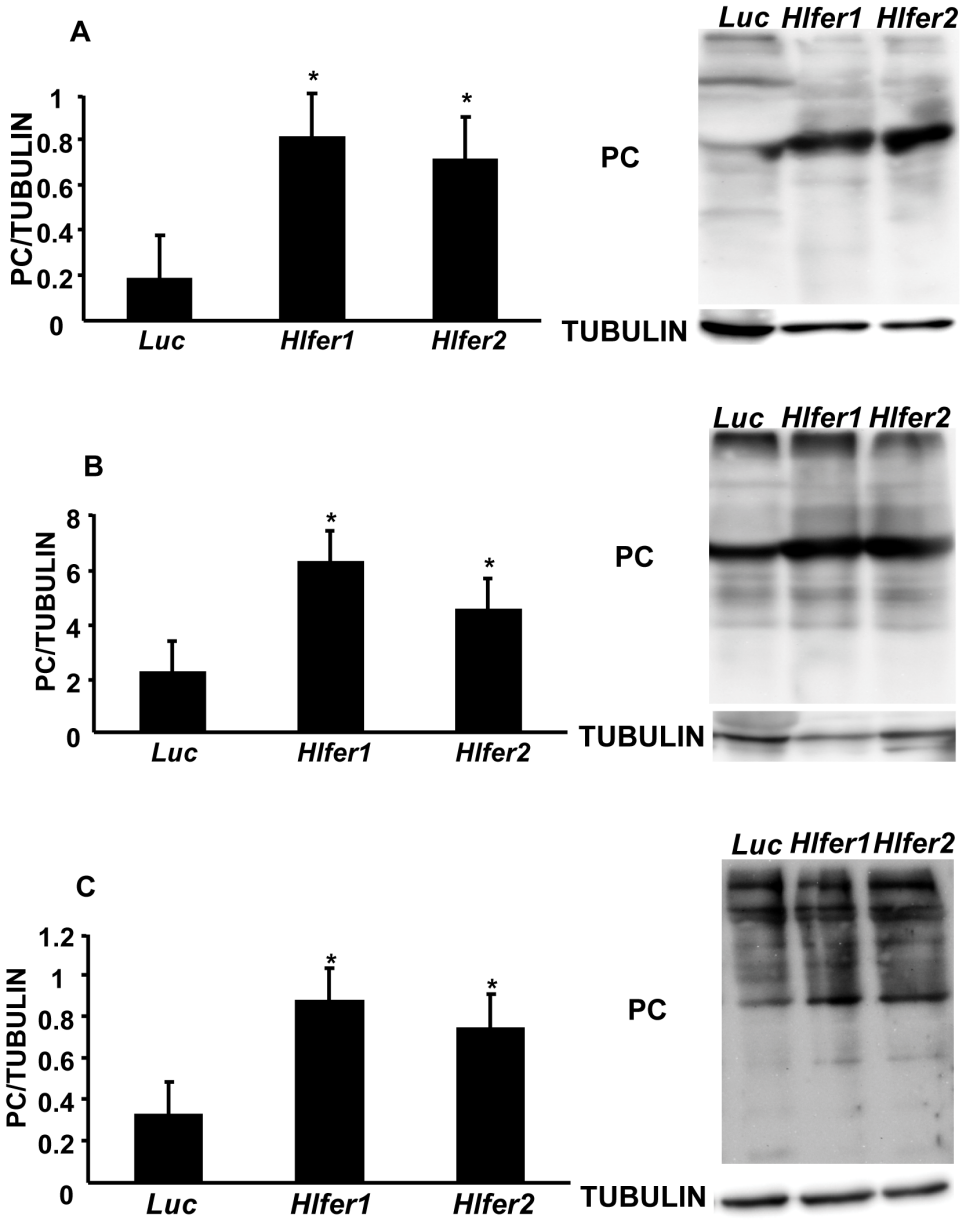
Detection of protein carbonyl groups (PC) from *Hlfer*-silenced ticks after blood feeding or injection of FAC. Total protein was extracted from whole ticks (A) and midguts (B) after blood feeding and whole ticks 72 h after injection of 100 µM FAC (C). After the transfer of proteins to a membrane through Western blot, the membrane was incubated first with dinitrophenylhydrazine (DNPH) for pre-derivitazation of the carbonyl group, before incubation with a specific anti-DNP antibody. Tubulin was used as internal control. The relative content of PC (strongest band) to tubulin was calculated after band densitometry analysis. Both *Hlfer1*- and *Hlfer2*-silenced ticks had significantly higher PC levels compared to the control (*Luc*) group. Data represent the means of three independent trials ± SE. *P<0.05, significantly different vs. *Luc*, Student's *t*-test.

Immunodetection using specific antibodies against MDA (Fig. 6) and PC (Fig. 7) showed that *Hlfer*-silenced ticks have significantly higher (*P*<0.05) levels of these oxidative stress biomarkers than the control group after blood feeding or FAC injection. Band densitometry analysis was performed to calculate the relative MDA or PC content of the samples based on tubulin. *Hlfer1*-silenced ticks showed the highest levels of MDA and PC, including engorged whole ticks and midguts and unfed ticks injected with FAC.

**Figure 8:**
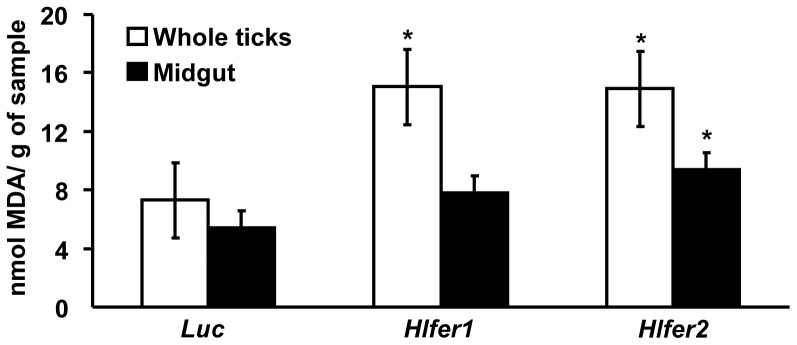
Thiobarbituric acid reactive species (TBARS) assay for *Hlfer*-silenced ticks after blood feeding. Whole bodies or midguts of ticks injected with *Hlfer1*, *Hlfer2*, or *Luc* dsRNA were collected after dropping from the host. Individual whole ticks or pooled midguts were weighed before being homogenized. After ultrasonication, the supernatants were obtained and boiled with TBARS reagent. Upon cooling and centrifugation, the absorbance of the supernatants was measured at OD_532_. The relative amount of MDA was calculated based on the sample weight and expressed as nmol/g. Both *Hlfer1*- and *Hlfer2*-silenced ticks had higher MDA levels in both whole ticks or midguts than the control (*Luc*) ticks. Values are means of 30 samples for each group ± SE. *P<0.05, significantly different vs. control, Student's *t-*test.

The level of MDA in *Hlfer*-silenced ticks after blood feeding was further evaluated using the TBARS assay, the most common technique employed in studying lipid peroxidation and oxidative damage [3]. The results showed that lipid peroxidation was higher in both *Hlfer*-silenced groups as compared to the *Luc*-injected control group, either in whole ticks or in midguts (Fig. 8). The highest level of MDA was observed in *Hlfer1*- and *Hlfer2*-silenced groups in whole ticks and midguts, respectively.

### *Hlfer1*-silenced ticks did not accumulate ferric iron after blood feeding or injection of FAC

**Figure 9:**
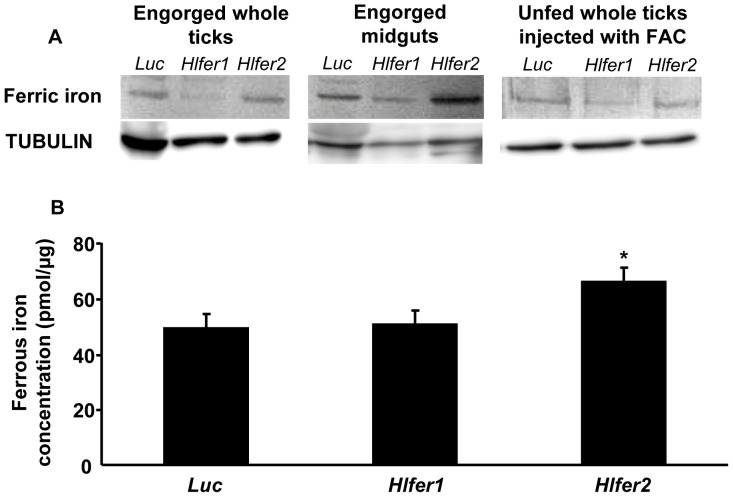
Evaluation of iron accumulation in *Hlfer*-silenced ticks. Ferric iron accumulation was evaluated by staining HlFER on native PAGE (A). Total protein was extracted from whole bodies and midguts after blood feeding and whole bodies 72 h after FAC injection. The amount of protein was adjusted based on the tubulin profile after Western blotting. Weak staining was observed in *Hlfer1*-silenced ticks. Ferrozine assay for ferrous iron 72 h after injection of 100 µM FAC to unfed *Hlfer*-silenced ticks. (B). Ten ticks from each group were homogenized and total protein concentration was measured. Ferrous iron was extracted with concentrated HCl and then detected using ferrozine. Absorbance was measured at OD_550_. Relative ferrous iron content was calculated based on protein concentration. *Hlfer2*-silenced ticks had significantly higher ferrous iron content than the control (*Luc*) group. *P<0.05, significantly different vs. *Luc*, Student's *t*-test.

Iron is stored in ferritin as ferric iron. We hypothesized that ferric iron accumulation should be reduced in the *Hlfer*-silenced ticks after blood feeding or FAC injection. To evaluate this hypothesis, staining of ferric iron after native PAGE was performed. Protein concentration was adjusted based on the tubulin level. In whole ticks and midguts after blood feeding, as well as in whole ticks injected with FAC, *Hlfer1*-silenced ticks weakly stained for ferric iron (Fig. 9A). Interestingly, the *Hlfer2*-silenced group still showed strong staining.

### Higher level of ferrous iron was detected in *Hlfer2*-silenced ticks injected with FAC

We also hypothesized that in the absence of HlFER, ferrous iron cannot be stored as ferric iron and should accumulate. Thus, the ferrozine assay for measuring non-heme iron was performed to determine the amount of ferrous iron in whole ticks injected with FAC after *Hlfer* knockdown [19], [20]. In performing the ferrozine assay, the addition of ascorbic acid was omitted to avoid the reduction of ferric to ferrous iron. The ferrozine assay showed that *Hlfer1*-silenced ticks had only a slightly higher ferrous iron level, while *Hlfer2*-silenced ticks had a significantly higher (*P*<0.05) ferrous iron level 72 h after FAC injection than the control group (Fig. 9B). HlFER2 being abundant in the hemolymph, this result suggests that knockdown of *Hlfer2* may have caused the accumulation of ferrous iron in the hemolymph of ticks.

## Discussion

Ticks are known for their ability to ingest large amounts of blood from their host, reaching more than a hundred times their unfed body weight. The numerous bioactive molecules in their saliva allow them to evade the host's immune and hemostatic mechanisms, which is important for successful attachment and feeding [27]. However, they also must cope with potentially toxic molecules in the host blood, including iron. Ferritin is an iron-storage protein involved in iron homeostasis in most living organisms. The physiological importance of ferritin in blood feeding and reproduction of the hard ticks *I. ricinus* [12] and *H. longicornis* [13] has been demonstrated through RNAi; however, the specific role of tick ferritins has not been demonstrated. In this study, we showed that *H. longicornis* ferritins act as antioxidant molecules that minimize oxidative stress.

Aside from the effects of *Hlfer* silencing on blood feeding and reproduction, we also previously reported that *Hlfer*-silenced ticks had high mortality after blood feeding. We showed here that this mortality is related to the iron-storage function of ferritin. For the first time, we exposed the ticks to exogenous iron by injecting FAC into the hemocoel. The silencing of *Hlfer* alone (data not shown) or with injection of water in unfed adult female ticks did not result in any mortality; however, mortality increased with each day after FAC injection. The group injected with *Hlfer2* dsRNA showed a more rapid increase in mortality and a lower survival rate at the end of the observation period compared to both *Hlfer1*-silenced and *Luc*-injected control groups. The injection of FAC introduced high levels of free iron in the hemocoel. Only the secretory HlFER2 is present in the tick's hemolymph. After the knockdown of *Hlfer2*, excessive ferrous iron, as we have demonstrated through the ferrozine assay, could have caused oxidative damage in the ticks that eventually lead to mortality. Conversely, the absence of HlFER1 after its knockdown could have led to high levels of intracellular ferrous iron.

To confirm that the mortality after FAC injection in *Hlfer*-silenced ticks is related to ferritin function, we performed additional experiments after injecting FAC into normal unfed adult ticks, including RT-PCR, Western blotting and IFAT. In contrast to reports of ferritin up-regulation on mosquitoes following artificial feeding and *in vitro* exposure of cells to iron [21], [22], [23], and after injection of iron in *Macrobrachium rosenbergii* [24] and *Bombus ignitus* [25], the transcript level of either *Hlfer* did not change in response to iron injection. Meanwhile, Western blot analyses showed an increasing protein level in whole ticks, particularly of HlFER1, in a time-dependent manner after FAC injection. In agreement with our previous conclusion, these results demonstrated the translational regulation of HlFER1 through the binding of the iron-responsive element (IRE) to the iron-regulatory protein (IRP) [28].

Interestingly, Western blot analysis of different organs showed that FAC injection stimulated the expression of both HlFER1 and HlFER2 in the midgut but not in the salivary glands or ovary. IFAT also showed the extensive fluorescence of digestive cells for HlFER1 after FAC injection, extending from the basal lamina to the cells close to the lumen, whereas HlFER2 was strong particularly along the basal lamina in the midgut. In contrast, no fluorescence was found in the salivary glands. These results suggest that the iron in the hemolymph may cross the basal lamina of the midgut for storage in HlFER1 of digestive cells. In mammals, circulating iron bound to transferrin can enter the basolateral membrane of enterocytes through transferrin receptor 1 [1]. However in the ticks, the function of transferrin in iron metabolism remains to be elucidated. Meanwhile, the increased fluorescence of HlFER2 along the basal lamina of the midgut after FAC injection may imply that iron in the hemolymph stimulated its expression with subsequent secretion, since the HlFER2 level in the hemolymph also increased after FAC injection. In mosquitoes, iron treatment resulted in an increase in the secretion of ferritin [22]. Here, since a high level of iron was present in the hemolymph, HlFER2 could have been secreted to sequester iron. Moreover, we previously concluded that HlFER2 is secreted from the midgut to remove iron and distribute it to other organs of the tick, in agreement with the model of iron metabolism in ticks proposed by Hajdusek et al. [12]. Presently, the other components of iron metabolism in ticks, as well as the regulatory signals in iron distribution, remain to be elucidated. Iron traffic during blood feeding in ticks must be systemically regulated, involving complex signal pathways. Whereas a series of signal pathways are known to be involved in iron traffic aside from the iron-binding proteins in mammals [29] and several proteins have already been identified in arthropods such as *Drosophila melanogaster* [30] and *Anopheles gambiae* [31], to function in iron absorption, these aspects require further investigations in the ticks.

The Prussian blue staining for ferric iron in native HlFER after native PAGE was useful in the assessment of ferric iron accumulation. We found increased staining after FAC injection, which may reflect the increased HlFER level and iron uptake of HlFER molecules at these time points. The increased ferric iron accumulation, together with the increased levels of both HlFERs in the midgut we mentioned earlier, supports our previous conclusion that the midgut is the primary organ for iron metabolism, most likely being the first organ exposed to large amounts of iron during blood feeding. Interestingly, ferric iron staining was weakened after *Hlfer1*-silencing but not after the silencing of *Hlfer2*. We previously found that HlFER1 was still expressed after *Hlfer2* silencing, particularly in the midgut [13]. Thus, the present result on ferric iron staining in *Hlfer2*-silenced ticks implicates HlFER1.

Iron is known to promote the formation of reactive oxygen species that can result in damage to macromolecules, including DNA, proteins and lipids—the condition collectively termed oxidative stress [2]. Iron was particularly reported to induce lipid peroxidation [32], [33] and oxidation of several amino acid residues in proteins [26]. Thus, the function of ferritin as a repository for excess iron is crucial to preventing oxidative damage. Here we showed that the knockdown of either *Hlfer* resulted in oxidative stress in ticks exposed to high levels of iron, either from blood meal or FAC injection. Similar to our previous study, *Hlfer*-silenced ticks infested on rabbits failed to engorge, weighing less than half of the *Luc*-injected ticks' engorged body weight, meaning they ingested a lower amount of blood. Oxidative stress was confirmed by the detection of malondialdehyde and protein carbonyl, which are products of lipid peroxidation and protein oxidation, respectively, and observation of higher levels in *Hlfer1*- and *Hlfer2*-silenced ticks than in *Luc*-injected ticks after blood feeding or injection of FAC. The TBARS assay was also employed to assess lipid peroxidation after blood feeding and similarly, it showed that *Hlfer*-silenced ticks had a higher degree of lipid peroxidation compared to the control. We also attempted to perform TBARS assay on unfed *Hlfer*-silenced ticks injected with FAC but due to the low sensitivity of this test [34], we were unable to detect the presence of MDA on the samples. Taken together, these results imply that without HlFER1 or HlFER2, free iron predisposed the ticks to oxidative stress that led to death.

We previously found abnormalities in the digestive cell morphology in *Hlfer*-silenced ticks during blood feeding, including altered shape, disrupted microvilli and cell membrane and vacuolated cytoplasm [13]. We hypothesized that these abnormalities resulted from oxidative damage. Here we show that the midgut of *Hlfer*-silenced ticks had high levels of MDA and protein carbonyl. Lipid peroxidation leads to alterations of biological membranes and gives rise to several products that are known to induce diverse biological effects [35]. MDA, which is one of the most known and most studied products of lipid peroxidation, is highly toxic and can interact with DNA and proteins and thus can impair physiological functions [36]. Aside from the direct injury caused by reactive oxygen species, products of lipid peroxidation including MDA can promote further injury. Protein carbonylation is another hallmark of oxidative stress resulting from irreversible oxidative modification of proteins that can be induced by transition metals including iron, ROS, products of lipid peroxidation including MDA, and glycoxidation. Protein carbonyls cannot be repaired and accumulation may lead to cell death [26]. Our present results also show that the midguts from *Hlfer1*-silenced ticks had the highest level of either oxidative stress biomarker, which corresponds to our previous observation of more severe abnormalities in the digestive cells of *Hlfer1*-silenced ticks. Moreover, we also previously reported a decrease in hematin production after *Hlfer1* silencing, indicative of impaired digestive activity. Oxidative stress can also alter physiological processes including heme detoxification in the midgut [37].

Several antioxidant enzymes that counteract reactive oxygen species, such as superoxide dismutase, glutathione S-transferase and thioredoxin, have been identified in hard ticks [38], [39]. These enzymes prevent oxidative stress by keeping the level of free radicals to a minimum. In the hard tick *Dermacentor variabilis*, these antioxidant enzymes were found in the midgut at day 6 of blood feeding, corresponding to the rapid feeding stage [38]. In this study, we demonstrated that, by sequestering ferrous iron and keeping it in the oxidized ferric form, ferritin is also an important antioxidant molecule in the hard tick because it prevents oxidative stress.

In summary, the silencing of two ferritin genes in the hard tick *H. longicornis* resulted to increased levels of oxidative stress biomarkers after a blood meal or injection of iron. Our results provide evidence for the first time that two kinds of ferritin act as antioxidant molecules in a hard tick that prevent oxidative stress during blood feeding, thus ensuring tick survival. This paper provides a clearer explanation on the crucial importance of ferritin in the ticks that we reported in our previous paper on *H. longicornis* [13], and also the other work in another hard tick, *I. ricinus* [12]. Moreover, our iron-injection experiment, which to our knowledge is employed for the first time in ticks, demonstrated that iron in the hemocoel can stimulate HlFER expression of the midgut and that iron molecules can be apparently transported from the hemolymph to digestive cells. However, further experiments are needed to elucidate this aspect of iron transport mechanism in ticks. Moreover, the iron-sequestration function of ferritin is implicated in immune response in many organisms [6]; thus, we are interested in the possible role of HlFERs in the tick immunity. Together with our previous results, our present study shows that ferritin is an important protective antigen of ticks that can be utilized to design a control strategy.

## Supporting Information

Figure S1 **Survival rate of** ***Hlfer*** **-silenced ticks after injection of sterilized high-purity water.** Four days after injection of *Hlfer1*, *Hlfer2*, or *Luciferase* dsRNA, sterilized high-purity was injected, and mortality was monitored. Low mortality was observed from all the three groups. n = 25 ticks per group. Bars represent standard error.
(TIF)

Figure S2 **Transcription profile of** ***H. longicornis*** **adult ticks injected with FAC.** Unfed adult ticks were injected with 50 µM or 100 µM FAC. Sterilized high-purity water was injected into the control group (0 µM). Total RNA was extracted from whole ticks at 24 h and 72 h after injection and RT-PCR analysis was performed using specific primers for *Hlfer1* and *Hlfer2*. cDNA was adjusted based on control amplification for *Hlactin*. No significant difference was observed among groups.
(TIF)

Figure S3 **Protein expression of** ***H. longicornis*** **ferritins in salivary glands of unfed ticks injected with different concentrations of FAC.** Salivary glands were collected from ticks at 24 h and 72 h after injection of 50 µM or 100 µM FAC. Sterilized high-purity water was injected into the control group (0 µM). Western blot analysis was performed using specific primary antibodies against *H. longicornis* FER1 (HlFER1) or *H. longicornis* FER2 (HlFER2). Tubulin was used as an internal control. No significant difference was observed among groups.
(TIF)

Figure S4 **Coomassie blue staining and Western blot analysis after native PAGE.** To further confirm that the bands stained for ferric iron from tick protein samples were HlFER, we performed Coomassie blue staining and Western blot analysis using specific anti-HlFER sera. (A) Coomassie blue staining showed all the bands of high molecular weight marker (M), the commercially prepared horse holoferritin (HF) and the tick protein (T). The weak band of approximately 440 kDa in the tick protein sample was presumed to be ferritin. Western blot analyses for HlFER1 (B) and HlFER2 (C) showed a single band of approximately 440 kDa. Arrow indicates the 440 kDa band in the high molecular marker while arrowheads point to tick ferritin.
(TIF)

Figure S5 **An IFAT examination of salivary glands 72 h after injection of different concentrations of FAC compared to control group injected with sterilized high-purity water.** Frozen sections of the salivary glands were incubated with specific mouse anti-HlFER1 or anti-HlFER2 sera. Normal mouse serum was used as negative control. Anti-mouse IgG conjugated with Alexa 594 was used as secondary antibody and nuclei were visualized using DAPI. No fluorescence was observed among groups. (Bars  = 20 µm).
(TIF)
