# Supplementary figures and images for: Two Kinds of Ferritin Protect Ixodid Ticks from Iron Overload and Consequent Oxidative Stress
Source: PLoS One. 2014 Mar 3;9(3):e90661. doi: 10.1371/journal.pone.0090661 (PMC3940913; doi:10.1371/journal.pone.0090661)

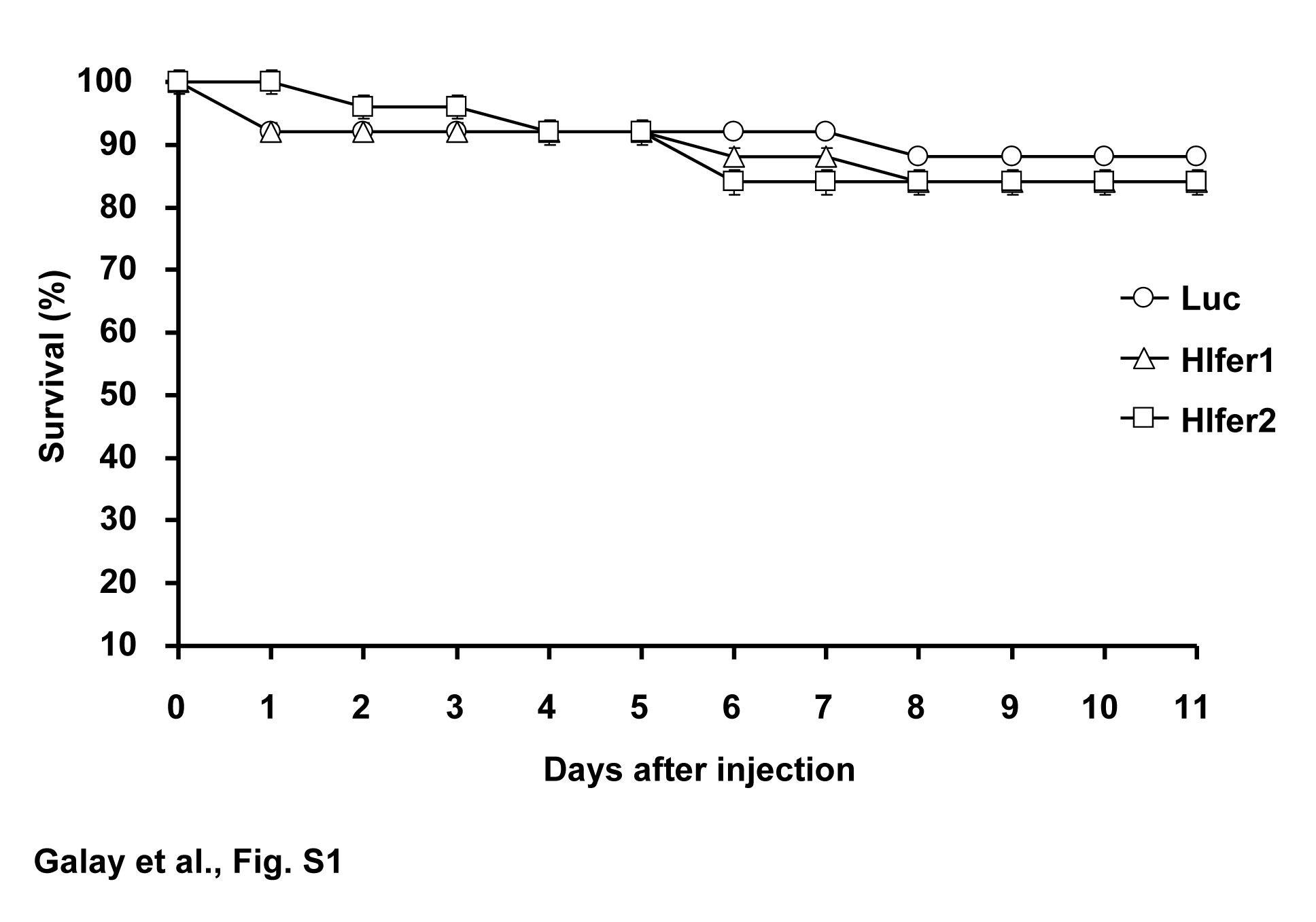

Supplement: Figure S1 — Survival rate of Hlfer -silenced ticks after injection of sterilized high-purity water. Four days after injection of Hlfer1, Hlfer2, or Luciferase dsRNA, sterilized high-purity was injected, and mortality was monitored. Low mortality was observed from all the three groups. n = 25 ticks per group. Bars represent standard error. (TIF) [file pone.0090661.s001.tif]

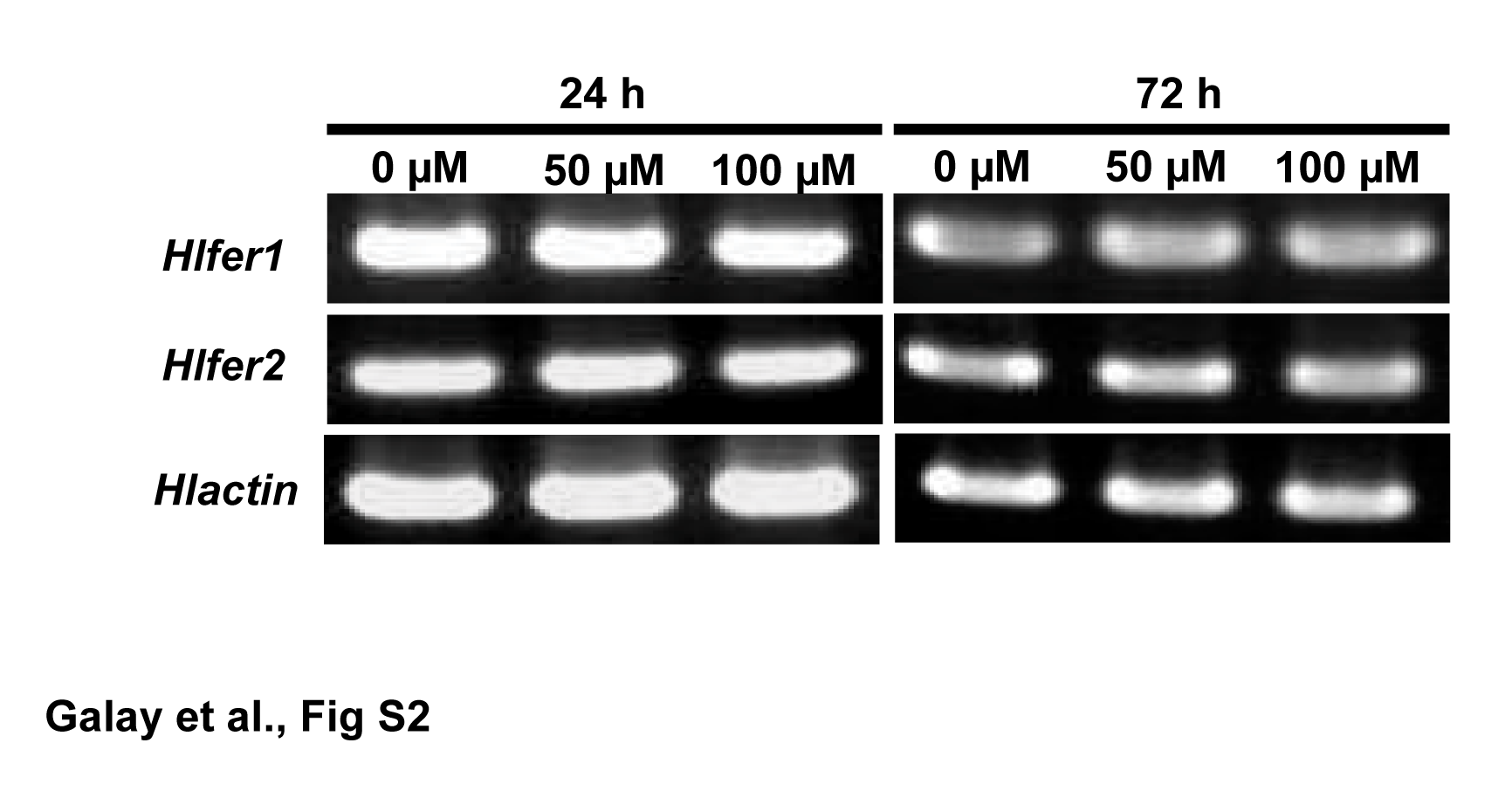

Supplement: Figure S2 — Transcription profile of H. longicornis adult ticks injected with FAC. Unfed adult ticks were injected with 50 µM or 100 µM FAC. Sterilized high-purity water was injected into the control group (0 µM). Total RNA was extracted from whole ticks at 24 h and 72 h after injection and RT-PCR analysis was performed using specific primers for Hlfer1 and Hlfer2. cDNA was adjusted based on control amplification for Hlactin. No significant difference was observed among groups. (TIF) [file pone.0090661.s002.tif]

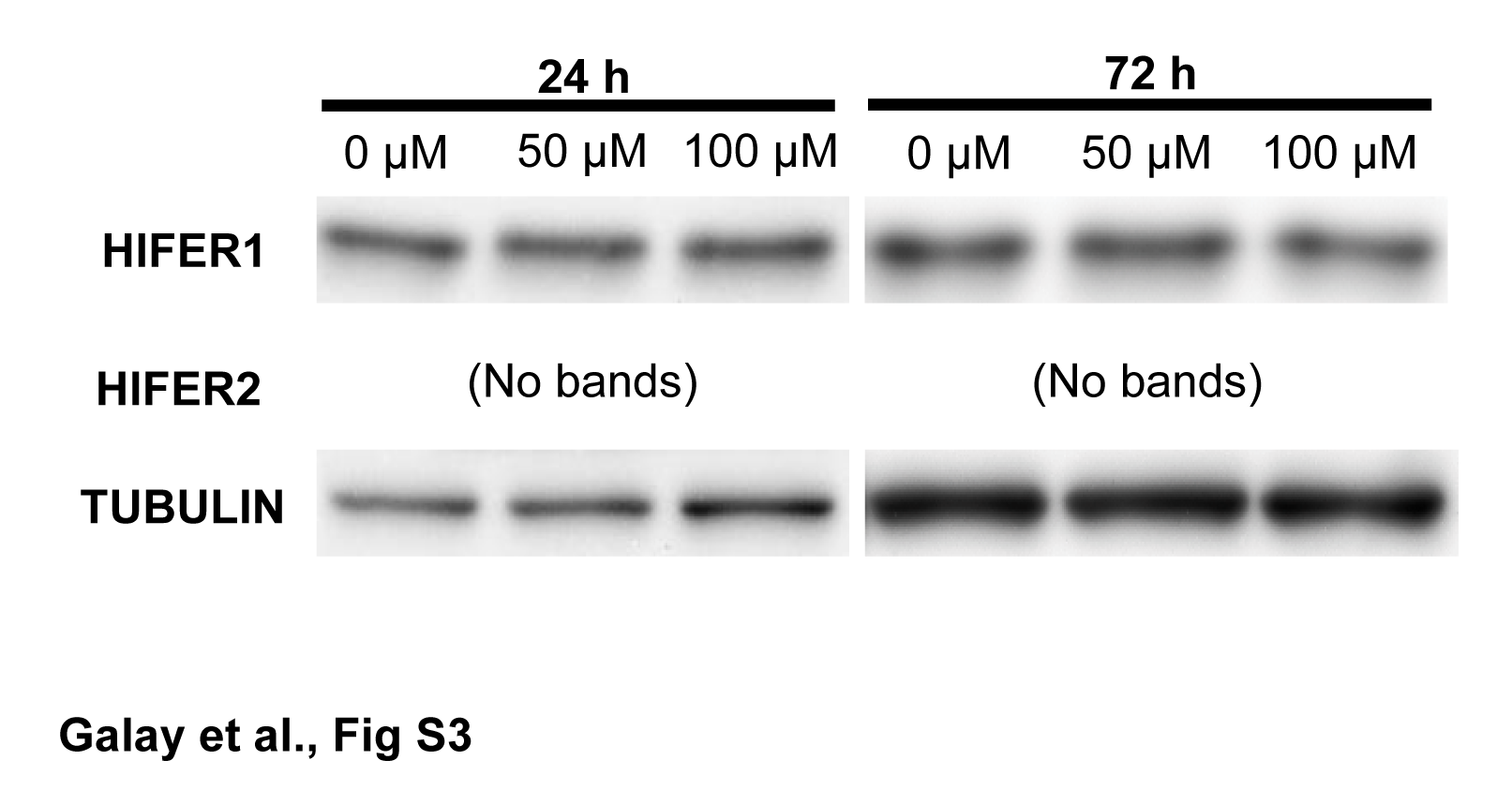

Supplement: Figure S3 — Protein expression of H. longicornis ferritins in salivary glands of unfed ticks injected with different concentrations of FAC. Salivary glands were collected from ticks at 24 h and 72 h after injection of 50 µM or 100 µM FAC. Sterilized high-purity water was injected into the control group (0 µM). Western blot analysis was performed using specific primary antibodies against H. longicornis FER1 (HlFER1) or H. longicornis FER2 (HlFER2). Tubulin was used as an internal control. No significant difference was observed among groups. (TIF) [file pone.0090661.s003.tif]

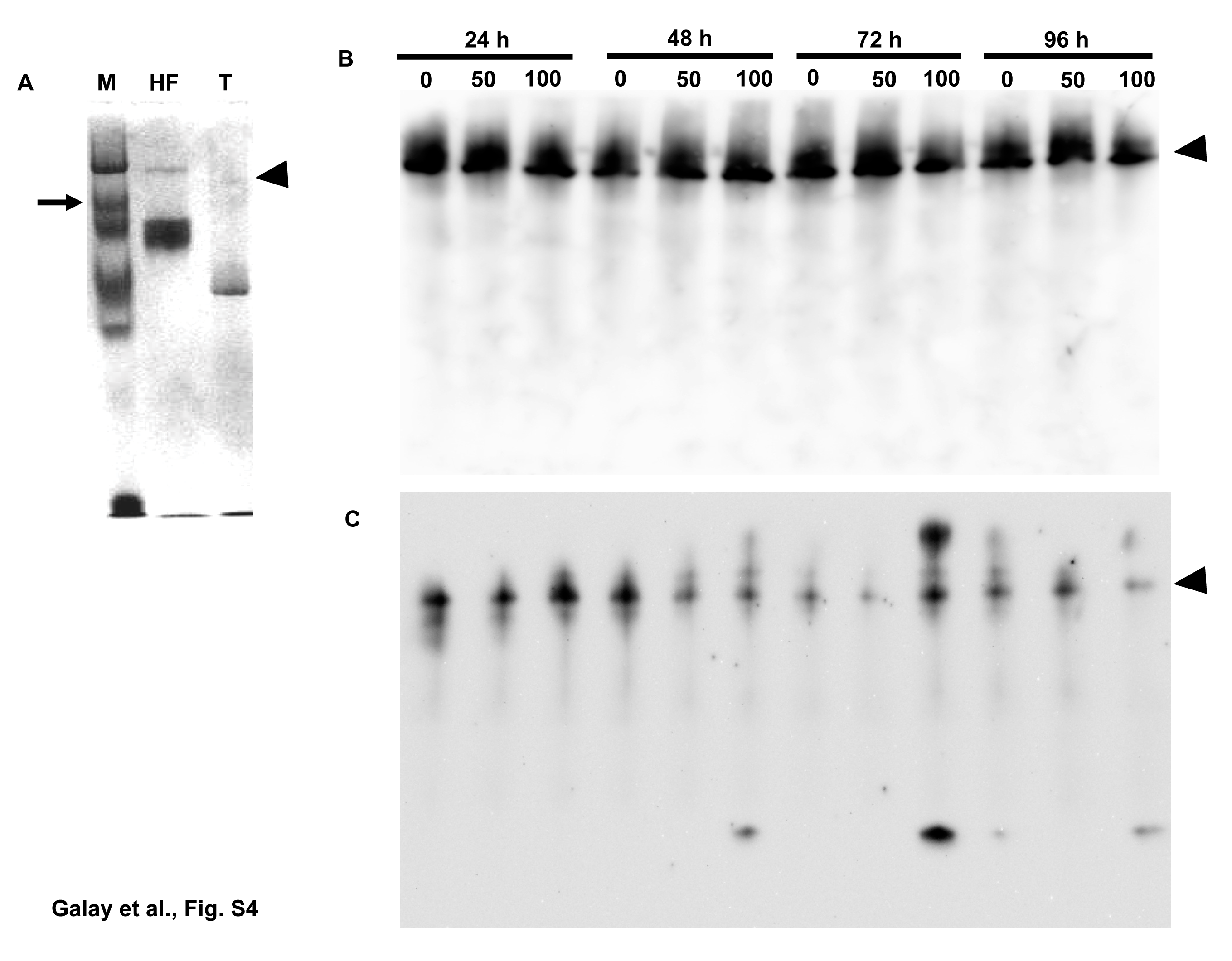

Supplement: Figure S4 — Coomassie blue staining and Western blot analysis after native PAGE. To further confirm that the bands stained for ferric iron from tick protein samples were HlFER, we performed Coomassie blue staining and Western blot analysis using specific anti-HlFER sera. (A) Coomassie blue staining showed all the bands of high molecular weight marker (M), the commercially prepared horse holoferritin (HF) and the tick protein (T). The weak band of approximately 440 kDa in the tick protein sample was presumed to be ferritin. Western blot analyses for HlFER1 (B) and HlFER2 (C) showed a single band of approximately 440 kDa. Arrow indicates the 440 kDa band in the high molecular marker while arrowheads point to tick ferritin. (TIF) [file pone.0090661.s004.tif]

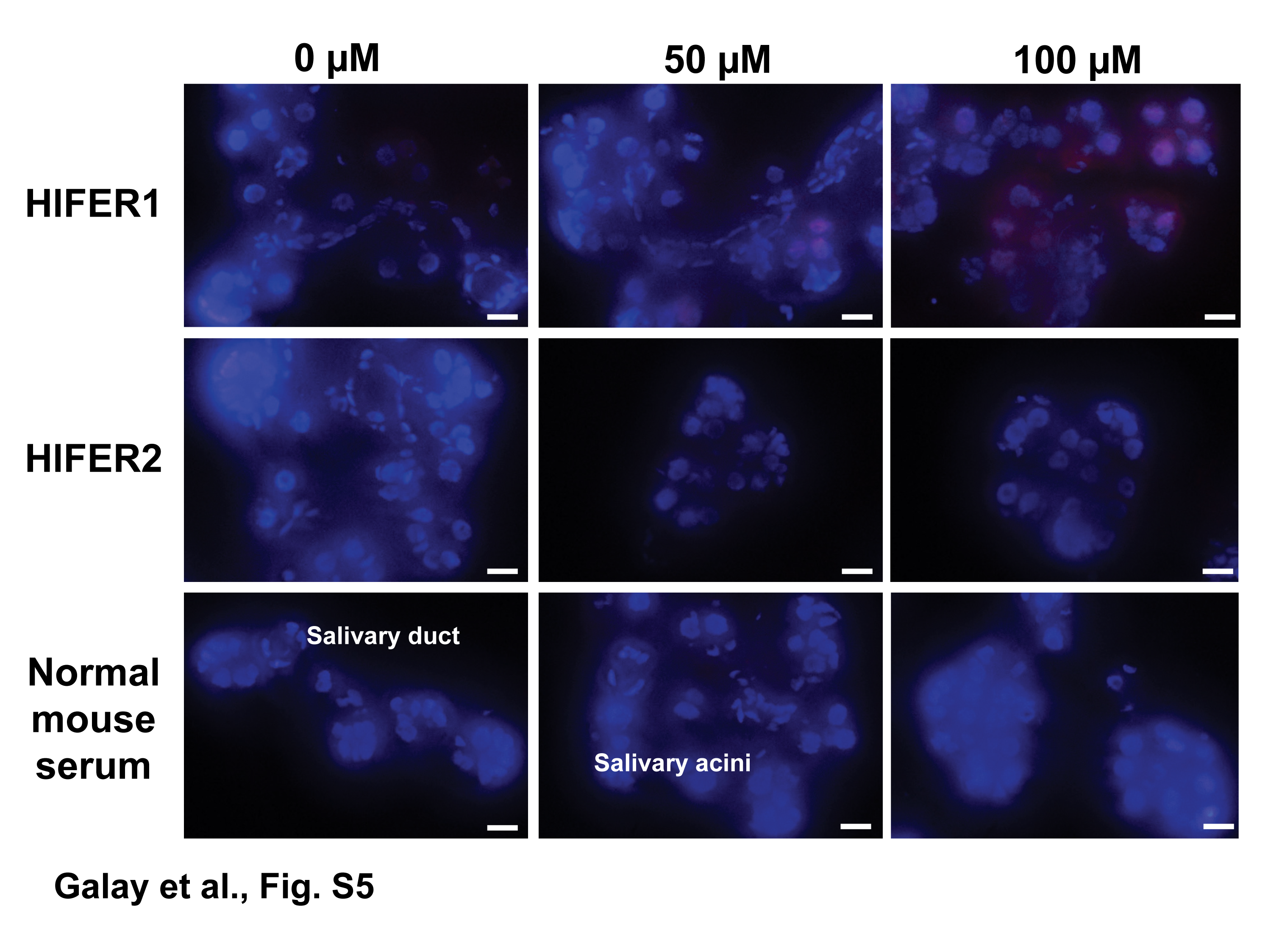

Supplement: Figure S5 — An IFAT examination of salivary glands 72 h after injection of different concentrations of FAC compared to control group injected with sterilized high-purity water. Frozen sections of the salivary glands were incubated with specific mouse anti-HlFER1 or anti-HlFER2 sera. Normal mouse serum was used as negative control. Anti-mouse IgG conjugated with Alexa 594 was used as secondary antibody and nuclei were visualized using DAPI. No fluorescence was observed among groups. (Bars = 20 µm). (TIF) [file pone.0090661.s005.tif]
